# ISSR Marker Based Population Genetic Study of* Melocanna baccifera* (Roxb.) Kurz: A Commercially Important Bamboo of Manipur, North-East India

**DOI:** 10.1155/2017/3757238

**Published:** 2017-01-10

**Authors:** Heikrujam Nilkanta, Thoungamba Amom, Leimapokpam Tikendra, Hamidur Rahaman, Potshangbam Nongdam

**Affiliations:** Department of Biotechnology, Manipur University, Canchipur, Imphal, Manipur 795003, India

## Abstract

*Melocanna baccifera* (Roxb.) Kurz is an economically important bamboo of North-East India experiencing population depletion in its natural habitats. Genetic variation studies were conducted in 7 populations sampled from 5 districts of Manipur using ISSR molecular markers. The investigation was carried out as a primary step towards developing effective conservation strategies for the protection of bamboo germplasm. ISSR marker analysis showed significant level of genetic variation within the populations as revealed by moderately high average values of Nei's genetic diversity (*H* 0.1639), Shannon's diversity index (*I* 0.2563), percentage of polymorphic bands (PPB 59.18), total genetic variation (*H*_t_ 0.1961), and genetic diversity within population (*H*_s_ 0.1639). The study also divulged a high genetic variation at species level with Shannon's diversity index (*I*), Nei's genetic diversity (*H*), and percentage of polymorphic band (PPB%) recorded at 0.3218, 0.1939, and 88.37, respectively. Genetic differentiation among the populations (*G*_st_) was merely 19.42% leaving 80.58% of genetic variation exhibited within the populations. The low genetic diversity between populations was consistent with AMOVA. The low genetic differentiation among populations coupled with existence of significantly high genetic diversity at species level indicated the urgent necessity of preserving and protecting all the existing natural bamboo populations in the region.

## 1. Introduction

Bamboos are multipurpose forest tree grasses having more than 1000 different uses [1]. They are extensively used as raw materials in paper and handicraft industries, in house construction, furniture making, water pipes, storage vessels, and other important household items [2, 3].* Melocanna baccifera* (Roxb.) Kurz, an evergreen arborescent, non-clump forming bamboo, is a dominant species of Manipur with its multiple uses in making of houses, furniture, handicraft, and other household items [4, 5]. This bamboo also provides important support for local ecosystem and is a priority species involved in protection of local soils and biodiversity of forested regions [6]. Overexploitation of bamboo by locals in its natural reserves may lead to dramatic fall of population. This will result in great environmental degradation due to water loss, soil erosion, and decline in natural biodiversity. Therefore, systematic management of depleting bamboo resource through the adoption of proper conservation strategies with either in situ or ex situ measures is the need of hour in the region. Assessing the level of genetic variation within and among natural bamboo populations is highly crucial for the development of effective conservation methods [7–10]. This is because the ability of a particular plant species to adapt effectively to changing environmental conditions depends on the level of genetic variability it possesses [11, 12]. DNA molecular markers like random amplified polymorphic DNA (RAPD), inter-simple sequence repeats (ISSR), amplified fragment length polymorphism (AFLP), and simple sequence repeat (SSR) have been used as influential molecular tools for determining genetic variation at species or population level in different plants [13–18]. The traditional approaches of employing morphological and vegetative characters for bamboo species identification and genetic variation studies had many shortcomings [19–21]. However, the application of RAPD, ISSR, AFLP, SSR, expressed sequence tag derived-simple sequence repeat (EST-SSR), sequence related amplified polymorphism (SRAP), and restriction fragment length polymorphism (RFLP) has enabled successful investigation of genetic variability in different bamboos [21–29]. ISSR molecular markers are widely used for population genetic analysis of different plants generating more reliable and reproducible bands than RAPD [30, 31]. They are technically simpler as compared to RFLP, SSR, and AFLP markers as no previous sequence information is required for generating DNA amplification products [32–34]. There have been limited studies on the genetic variation of* M. baccifera* using ISSR markers and no reports are available on the population genetic studies of the bamboo in Manipur. The present study aimed to investigate the genetic diversity and population genetic structure of* M. baccifera *in 5 districts of Manipur using ISSR markers. This study could contribute to the development of strategies for effective conservation and sustainable utilization of this bamboo for ecological and economic gains by better understanding the genetic diversity profile at the species and population level.

## 2. Materials and Methods

### 2.1. Plant Materials and Population Sampling

**Figure 1:**
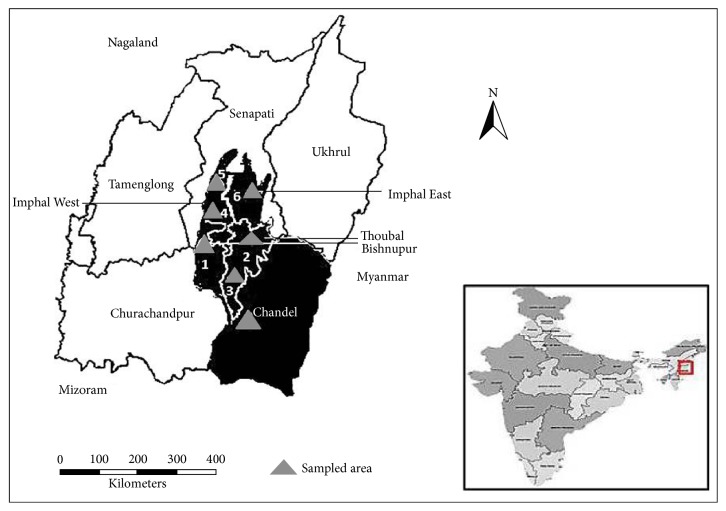
Distribution and location of 7 populations of* M. baccifera *sampled in 5 districts (blackened area) of Manipur.

A total of 93 individuals of* M. baccifera* representing 7 populations were collected from different locations spreading across 5 districts of Manipur in North-East India, namely, Bishnupur, Thoubal, Imphal West, Imphal East, and Chandel (Figure 1). The geographical location of each population and its code name and size were presented in Table 1. Fresh leaves were obtained from bamboo plants constituting a particular population. The sample collection was performed from individual plants separated by at least 50 m so as to prevent any possibility of sampling within the same clones. The leaf samples were then stored at −20°C until the DNA extraction was performed.

### 2.2. DNA Extraction and ISSR Amplification

The genomic DNA was extracted from the collected leaf samples using the CTAB method with slight modifications [35]. The leaves after being finely ground to fine powder in liquid nitrogen were mixed with freshly prepared CTAB extraction buffer and incubated at 50°C for 15–20 minutes in hot water bath before being subjected to centrifugation at 12000 rpm for 5 minutes. The resultant supernatant was treated with chloroform : isoamyl alcohol (24 : 1) followed by another centrifugation at 13000 rpm for 1-2 minutes. The pellet obtained after 7.5 M ammonium acetate treatment was washed several times with 70% ice-cold ethanol and dried before being resuspended in sterile DNase-free double distilled water. The DNA sample obtained was further incubated at 65°C for 20 minutes to destroy any DNase if present and stored at 4°C for subsequent analysis. DNA quality and quantity were determined through spectrophotometry at 260 and 280 nm, respectively. The purity and integrity were later checked by performing 1.0% agarose gel electrophoresis and comparing the intensity of the resultant bands with 1 kb DNA ladder (Hi-Media). The DNA samples were finally diluted to 50 ng/*μ*L and stored at −20°C for further use. The genetic profile study of* M. baccifera* was conducted by using 5 ISSR markers, namely, UBC-813, UBC-822, UBC-828, UBC-868, and UBC-878 obtained commercially from the University of British Columbia (Vancouver, Canada). The selected primers showed good, reliable, repetitive, and distinct bands which enabled effective scoring for genetic diversity study within and among the populations.

The DNA amplification mixture of 25 *μ*L contained 25 ng template DNA, 1x PCR buffer, 1.5 mM MgCl_2_, 200 mM dNTPs, 1 *μ*M primers (UBC-813, UBC-822, UBC-828, UBC-868, and UBC-878), and 0.6 U* Tag* DNA polymerase and double distilled sterile water. The PCR components were prepared as master mix for each primer to minimize the pipetting error. The amplification reaction was performed in a thermal cycler (Eppendorf Mastercycler nexus X2) with amplification cycle condition of initial 4 minutes' strands separation at 94°C followed by 40 cycles of 94°C for 45 secs, 53°C for 1 min, and 72°C for 2 minutes and final extension at 72°C for 10 minutes. The products obtained after PCR amplification were electrophoresed in 2% agarose gel in 0.5x TBE buffer at 100 V for around 3 hours and gel was stained with ethidium bromide (0.5 *μ*g/mL). The fragments after staining were visualized in gel documentation system (Alpha Innotech, USA). A 1000 bp DNA ladder (Hi-media) was used as a size marker for every gel run.

### 2.3. Data Analysis

Distinct, reproducible, well resolved fragments were scored as present (1) or absent (0) for each ISSR reaction and were displayed as part of a binary matrix. The data matrices obtained were analyzed using POPGENE version 1.31 [36]. Genetic parameters such as percentage of polymorphic band(s) (PPB), observed number of alleles (*N*_a_), the effective number of alleles (*N*_e_), Nei's genetic diversity (*H*), Shannon's information index (*I*), total genetic diversity (*H*_t_), genetic diversity within population (*H*_s_), Nei's genetic differentiation index among populations (*G*_st_), and gene flow estimates between populations (*N*_m_) were determined with POPGENE version 1.31 [36]. Genetic divergence between populations of the bamboo species was also investigated using Nie's unbiased genetic distances and genetic identities [37]. A study on the correlation (Mantel test) between genetic and geographic distance between the bamboo populations was done using TEPGA version 1.3 with 999 permutations [38]. An analysis of molecular variance (AMOVA) was performed to estimate the variance components and their significance levels of genetic variation within and among populations using GenALEx version 6.5 [39]. The unbiased genetic distance was utilized for the construction of a dendrogram using UPGMA (Unweighted Pair Group Arithmetic Mean Method) in POPGENE program version 1.31. In addition, Nei's genetic distance matrix was used to construct Neighbor-Joining dendrogram for the 93 individuals belonging to 7 different populations of* M. baccifera* using Mega 5.10 [40]. Principal coordinate analysis (PCoA) was performed using GenALEx version 6.5 [39] to determine relative genetic distance between individuals and to check the consistency of population genetic differentiation as defined by cluster analysis. The Bayesian model base clustering method of STRUCTURE software version 2.2 was employed to evaluate the genetic population structure and detect the gene pools contributing to bamboo germplasm collection [41]. Ancestry model with admixture and correlated allele frequency model were used to determine the posterior probability of the data and a burn in period of 10,000 was set followed by 10,000 Markov Chain Monte Carlo (MCMC) replications. The number of *K* was set from 1 to 8 with 10 independent runs for every *K* value. The results of the STRUCTURE outfile were utilized to determine the optimum *K* value following the simulation method of Evanno et al. [42] employing software STRUCTURE HARVESTER [43].

## 3. Results

### 3.1. Genetic Diversity

The five different ISSR markers amplified a total of 93 individuals representing 7 different populations of* M. baccifera *and generating high level of genetic polymorphism. High genetic variation at species level was observed in the present investigation with the recording of percentage of polymorphic bands (PPB) at 88.37%. The values for *N*_a_, *N*_e_, *H*, and *I* at species level were also recorded at 1.8837, 1.2751, 0.1939, and 0.3218, respectively, showing a relatively high level of genetic diversity (Table 2). However, the genetic differentiation at population level was relatively low as compared to genetic variation evidenced at species level. This was proved by moderate PPB (%) recorded in the range of 51.16% to 69.77% averaging at 59.18%. Similarly, the effective number of alleles (*N*_e_) varied from 1.1626 to 1.3923 with an average value of 1.2625, while the observed number of alleles (*N*_a_) ranged from 1.5116 to 1.6977 averaging at 1.5940. Nei's genetic diversity (*H*) also extended from 0.1149 to 0.2282 with an average of 0.1639, and Shannon's information index (*I*) spanned from 0.1927 to 0.3400 with an average value of 0.2563. THBL-A and CHDL exhibited highest genetic diversity at population level while the lowest variation was found in IMW-B.

### 3.2. Genetic Differentiation and Relationship among Populations

**Figure 2:**
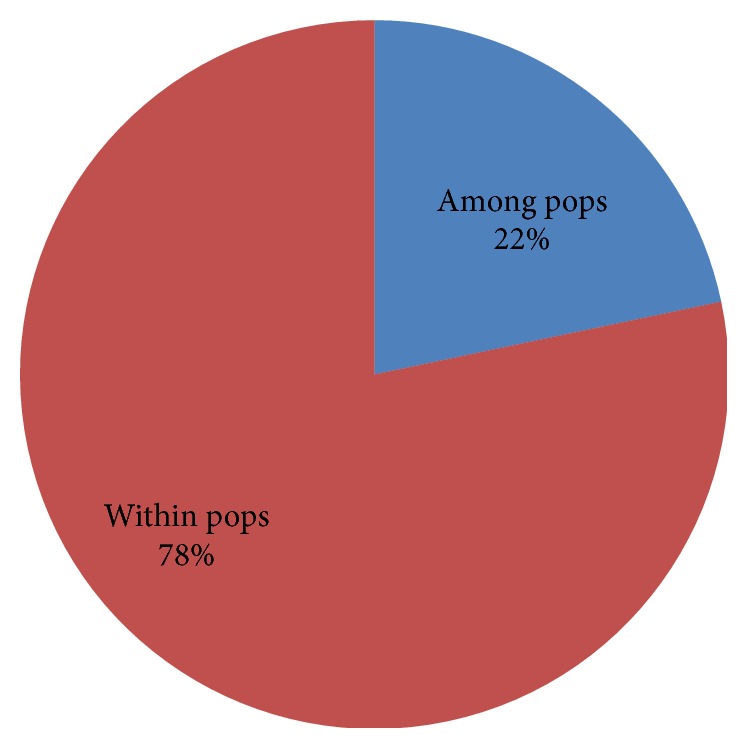
Analysis of molecular variance (AMOVA) revealed 22% of the genetic variation existing among populations and 78% within the populations.

The genetic diversity within population (*H*_s_) and the total genetic diversity (*H*_t_) of the species were recorded at 0.1639 and 0.1961, respectively. The observed genetic differentiation among populations (*G*_st_) was 0.1942 demonstrating the presence of 19.42% of genetic variation among the populations. The finding was consistent with the result of analysis of molecular variance (AMOVA) which revealed 22% of the genetic variation existing among populations and 78% within the populations (Figure 2). The differences exhibited between the populations were found to be highly significant (*P* = 0.001) (Table 3). The result was further substantiated by the existence of significantly high level of gene flow among the populations as shown by high estimate of *N*_m_ value (2.5455).

**Figure 3:**
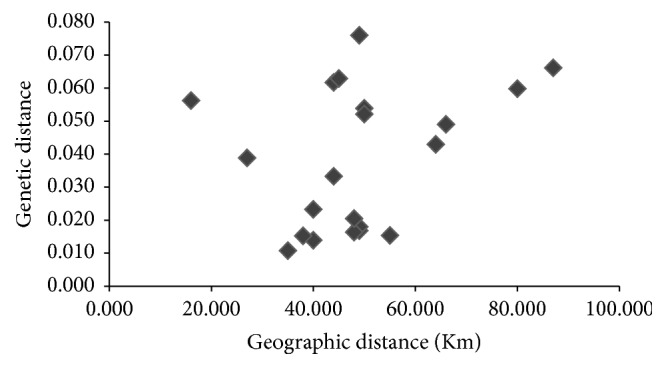
Correlation between genetic and geographic distance among 7 populations of* M. baccifera.*

**Figure 4:**
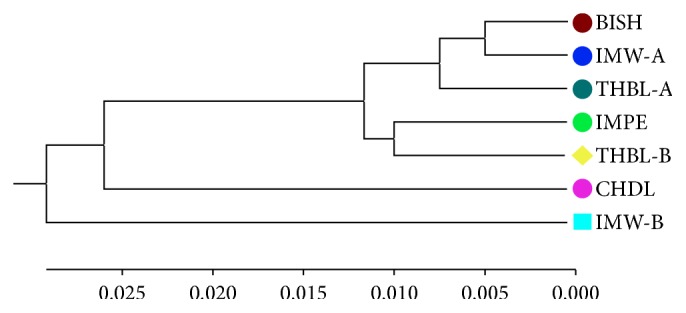
UPGMA dendrogram based on Nei's (1972) unbiased measures of genetic distance among 7 populations of* M. baccifera.*

**Figure 5:**
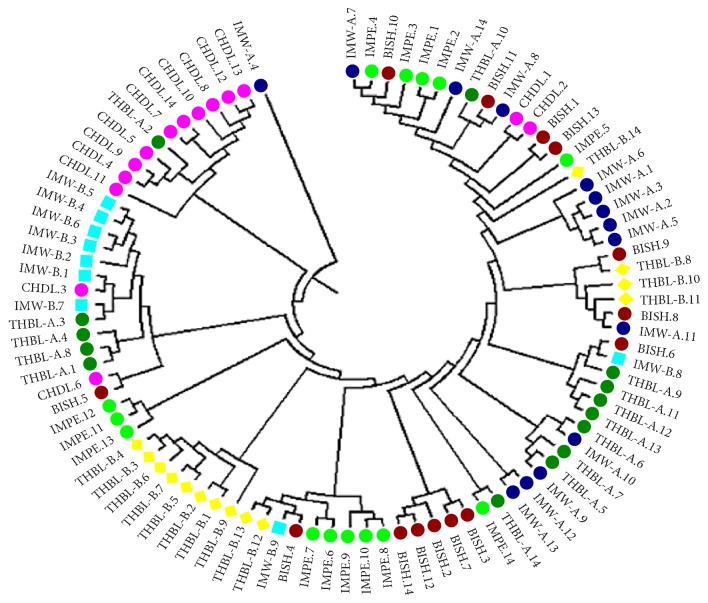
Neighbor-Joining dendrogram clustering pattern for 93 individuals belonging to 7 populations of* M. baccifera.*

Mantel test was performed to find out if there was any correlation between genetic relatedness between populations and geographic distance separating them. The test result indicated that there was no significant correlation between the genetic and geographic distance (*r* = 0.311; *P* = 0.240) (Figure 3). The genetic distance obtained between the 7 populations of* M. baccifera* varied from 0.011 between BISH and IMW-A to 0.076 between CHDL and THBL-B, respectively (Table 4). The dendrogram display based on Nei's [44] unbiased measures of genetic distances showed only one major cluster comprising of BISH, IMW-A, THBL-A, IMPE, CHDL, and THBL-B. The CHDL population tended to separate from the cluster indicating its genetic isolation from other populations in the group (Figure 4). But CHDL exhibited more genetic proximity to THBL-B as compared to other remaining populations in the same cluster with the recording of significantly high value of Nei's unbiased measures of genetic identity (0.967). The existing greater genetic identity between CHDL and THBL-B in spite of individuals of CHDL being sampled from hilly dominated region may be attributed to geographic closeness of the two populations. The lone IMW-B population diverged from the major cluster in the dendrogram depicting its less genetic relatedness to remaining populations existing in the major cluster. Neighbor-Joining radial tree obtained for the individual samples based on Nei's genetic distance showed most of the plants belonging to different population origins separated distinctly though some individuals were partly mixed clustered (Figure 5). Individual genotypes belonging to same population were more closely related genetically than those of other populations.

**Figure 6:**
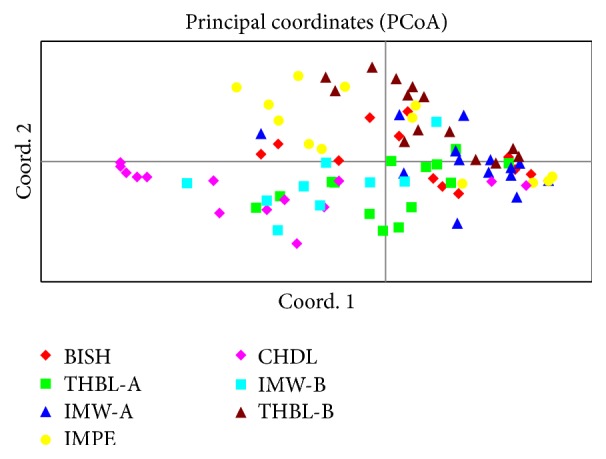
Two-dimensional plot of principal coordinate analysis (PCoA) showing clustering of individual samples belonging to 7 populations of* M. baccifera.*

The principal coordinate analysis (PCoA) was conducted to determine the spatial representation of genetic distances observed among individuals of different populations and also to check the consistency of population genetic differentiation as defined by cluster analysis. The two-dimensional PCoA plot showed the first principal coordinate accounting for 16.32% while the second coordinate produced 10.90% of the total genetic variation. The individuals of seven bamboo populations were distributed in the plot in accordance with the cluster analysis. The individuals belonging to CHDL and IMW-B were scattered separately from the rest of the populations (Figure 6). The remaining populations were clustered as one group with individuals more or less intermixed as was depicted in the cluster analysis.

**Figure 7:**
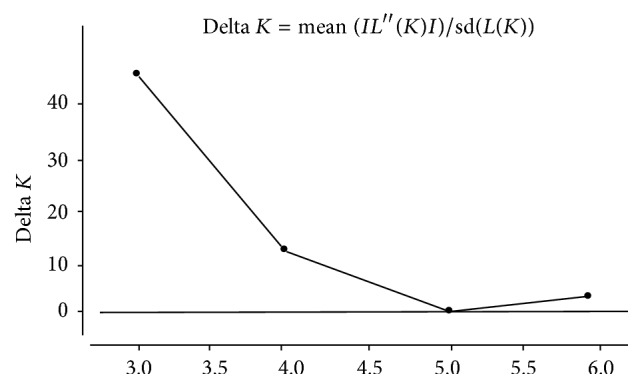
Result of Bayesian assignment analysis suggesting *K* = 3 as most likely number of clusters as delta *K* value maximum at *K* = 3.

**Figure 8:**
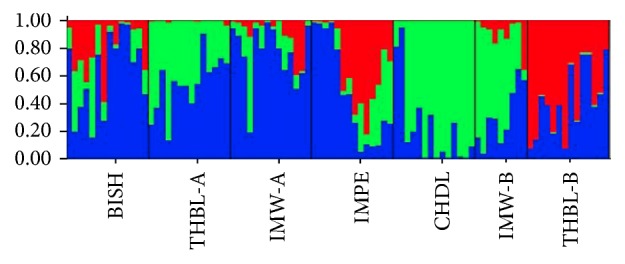
Population structure pattern of 7 populations of* M. baccifera *generated using STRUCTURE program when *K* = 3.

The highest delta *K* which determined the best value of *K* was obtained from STRUCTURE HARVESTER and was found to be shown at *K* = 3 (Figure 7). The result indicated that all the 93 individuals of seven populations of* M. baccifera* shared three genetic pools and were assigned into three different clusters (Figure 8). The seven populations displayed significant degree of mixed ancestry as represented in the structure diagram.

## 4. Discussion

The genetic variation analysis conducted in 7 populations of* M. baccifera* revealed the existence of high genetic diversity within population which was similar to earlier finding in* Dendrocalamus membranaceus *using ISSR markers [28]. Plants which are long lived, outcrossing, and self-incompatible have higher genetic variation at species level and lower differentiation among populations [11, 45]. The high genetic variation within the population of* M. baccifera *was expected as being a long lived woody bamboo with long vegetative phase [46]. Self-incompatibility is another important factor in maintaining high genetic variability in population [47] and* M. baccifera* is known to be self-incompatible and outcrossing bamboo thereby displaying significant genetic diversity within population. Nybom [48] also reported retention of most of genetic variability within populations of long lived and outcrossing plant species when many RAPD and sequence tagged microsatellite site (STMs) based analysis were performed. The plants with high geographical ranges tend to maintain higher genetic diversity than geographically localized species [49].* M. baccifera* is known to show diverse distribution pattern along the various geographical locations of Manipur [6]. Genetic diversity within populations is also influenced by many factors such as mating system, population size, extended time period with low number of individuals, genetic drift, and gene flow [50]. High genetic diversity within small populations can also be exhibited if reduction of population size had taken place very recently, especially when it occurred within a generation or two for the concerned species [51]. In such cases the surviving individuals are effectively sampled from the populations that have existed before. The existence of significant genetic variation within the populations of* M. baccifera* under study may also be due to sudden reduction in population size in short span of time. This presumption might hold true as more accessible individuals growing in the region have been exploited by locals for construction of houses and other household products along with the utilization of their tender shoots as popular vegetable items.

The coefficient of genetic differentiation among populations (*G*_st_) was 0.1942 which was higher than the mean value (*G*_st_ 0.073) observed for 121 woody species examined using allozyme markers [52]. Yang et al. [28] also recorded low *G*_st_ (0.252) in ISSR analysis of* Dendrocalamus membranaceus *populations in Yunnan province of China. The low *G*_st_ value recorded for* M. baccifera *indicated low level of genetic differentiation among the populations. Low genetic differentiation between populations had also been confirmed by AMOVA (analysis of molecular variance). The finding was also consistent with high *N*_m_ (2.5455) recorded which was much higher than *N*_m_ (0.0101) recorded for* Dendrocalamus giganteus* by Tian et al. [21]. The genetic differentiation between populations is negatively correlated with gene flow [53]. Gene flow between the populations might have been induced by pollens and seeds highly influencing plant evolution [54]. High gene flow (*N*_m_ 2.545) was observed in spite of poor seed dispersal mechanism of* M. baccifera*. The flower cycle of this bamboo is relatively long and large seeds which fall near the foot of plants are mostly eaten by rats and rodents. However,* M. baccifera* being one of the economically important bamboos of the region might have undergone human mediated movement of genotypes through local people, farmers, and entrepreneurs. This may lead to enhancement of gene flow among populations and produce overlapping and intermixing of bamboo genotypes from different populations. The easy genetic movement might also be possible as the regions of population sampling in the present study were confined mostly to plain valley area. In fact majority of the populations under study were located in valley districts except for the one population sampled from hill district of Chandel. The STRUCTURE result also revealed high degree of admixture among the 7 populations in three genetic clusters. The existence of great extent of admixture may be attributed to cultivation and utilization pattern of the bamboo species. Geographic isolation is another major factor influencing the genetic differentiation among populations by limiting the amount of gene flow via both pollen and seeds and also through human activities [55]. Theoretically gene flow of more than four migrants per generation is sufficient to prevent genetic differentiation between populations due to drift alone. In our study, the gene flow estimate was high (*N*_m_ 2.5455) ruling out the possibility of inducing the genetic differentiation among populations of* M. baccifera* due to geographical isolations. The results from the Mantel test also supported the above observation as it showed no correlation between the genetic and geographic distances between different populations. The ultimate goal of conservation is to ensure the continuous survival of population and to maintain their evolutionary potential by preserving natural levels of genetic diversity within and between populations [9, 56, 57]. The natural* M. baccifera* resources in the selected sites have been exploited by locals for many years leading to loss of plant resources, habitat destruction, and fragmentation. The dramatic fall of bamboo natural population due to increasing demand for its multiutility characters ensures that there is urgent requirement of effective conservation. The population size of* M. baccifera* sampled from different locations was small requiring population enhancement to maintain standard effective size for high genetic diversity preservation. Genetic drift in very small population might also cause rapid genetic erosion and increased the risk of extinction of the bamboo species. Considering the* M. baccifera* maintaining significant diversity at species level but low genetic variation among populations and that many native habitats and ecosystems are being destroyed by human interferences, great effort should be made to preserve individuals of the existing populations. Exploitation of bamboos and habitat destruction should be prohibited in the region till effective in situ and ex situ conservation strategies are established. This will prevent further reduction of population size and conserve the overall genetic base and structure of the bamboo species. The in situ efforts to conserve the remaining habitats should be combined with ex situ approach through seed and vegetative propagation of culm cutting, division, and tissue culture technology. These activities should be performed with a view to establishing a new generation of plants both cultivable and wild. A more effective protection strategy may also be framed especially for BISH, IMW-A, IMW-B, and THBL-B which displayed low genetic diversity at population level. Sensitization of locals about the prevailing genetic scenario of alarming bamboo populations and involvement of local communities in framing protection policies will be a highly effective approach to bamboo conservation. The presence of high genetic variation within populations of* M. baccifera *emphasizes the necessity of preserving and conserving all the existing 7 natural populations and their habitats in Manipur.

## Figures and Tables

**Table 1:** Location of sample collection and sample size for seven populations of *M. baccifera* in the present study.

Population code	Locality	Sample size	Latitude (N)	Longitude (E)	Elevation (m)
BISH	Bishnupur district	14	24°37′8.78′′	93°45′42.05′′	828
THBL-A	Areas covering right to Indo-Myanmar road, Thoubal district	14	24°34′7.93′′	94°2′0.86′′	790
IMW-A	Canchipur, Sekmai, Imphal West district	14	24°36′13.03′′	94°4′0.71′′	800
IMPE	Imphal East district	14	24°45′2.48′′	93°56′0.72′′	975
CHDL	Chandel district	14	24°50′9.81′′	93°54′51.22′′	1100
IMW-B	Kangchup areas, Imphal West district	9	24°47′32.38′′	94°2′18.63′′	790
THBL-B	Areas spanning forested regions to the left of Indo-Myanmar road, Thoubal district	14	24°27′10.64′′	94°1′33.04′′	1500

**Table 2:** Genetic diversity within populations and genetic differentiation parameters of seven populations of *M. baccifera*.

Population	*N*_a_	*N*_e_	*H*	*I*	PPB (%)		*H*_t_	*H*_s_	*G*_st_	*N*_m_
BISH	1.5581	1.1995	0.1320	0.2128	55.81					
THBL A	1.6977	1.2411	0.1547	0.2504	69.77					
IMW-A	1.5349	1.1626	0.1149	0.1927	53.49					
IMPE	1.6279	1.2618	0.1678	0.2651	62.79					
CHDL	1.6512	1.3923	0.2282	0.3400	65.12					
IMW-B	1.5116	1.2914	0.1720	0.2594	51.16					
THBL-B	1.5814	1.2888	0.1780	0.2737	58.14					
Average	1.5940	1.2625	0.1639	0.2563	59.18					
Species level	1.8837	1.2751	0.1939	0.3218	88.37	Total	0.1961	0.1639	0.1942	2.5455

*N*_a_, observed number of alleles; *N*_e_, effective number of alleles; *H*, Nei's gene diversity; *I*, Shannon's information indices; PPB, percentage of polymorphic bands; *H*_t_, total genetic diversity; *H*_s_, genetic diversity within populations; *G*_st_, the relative magnitude of genetic differentiation among populations; *N*_m_, estimate of gene flow among populations.

**Table 3:** Analysis of molecular variance (AMOVA) for five ISSR markers among *M. baccifera* populations.

Source of variations	Degree of freedom	Sum of squares	Mean square	Variance components	% of total variance	*P* value
Among populations	6	136.87	22.81	1.35	22.00	<0.001
Within populations	86	419.92	4.88	4.88	78.00	<0.001
*Total*	92	556.79		6.23	100	

**Table 4:** Nei's unbiased measures of genetic identity (above diagonal) and genetic distance (below diagonal) of the seven populations of *M. baccifera.*

BISH	THBL-A	IMW-A	IMPE	CHDL	IMW-B	THBL-B	Population
*∗∗∗∗∗∗*	0.986	0.989	0.985	0.958	0.948	0.984	BISH
0.014	*∗∗∗∗∗∗∗*	0.983	0.982	0.967	0.949	0.962	THBL-A
0.011	0.017	*∗∗∗∗∗∗∗*	0.985	0.942	0.945	0.977	IMW-A
0.015	0.018	0.015	*∗∗∗∗∗∗∗*	0.952	0.940	0.980	IMPE
0.043	0.033	0.060	0.049	*∗∗∗∗∗∗∗*	0.936	0.927	CHDL
0.054	0.052	0.056	0.062	0.066	*∗∗∗∗∗∗∗*	0.939	IMW-B
0.016	0.039	0.023	0.021	0.076	0.063	*∗∗∗∗∗∗∗*	THBL-B
